# Managing flash flood crises with cultural perspectives: A user-centric feature identification study

**DOI:** 10.1371/journal.pone.0318996

**Published:** 2025-02-13

**Authors:** Siti Fatimah Abdul Razak, Sumendra Yogarayan, Umar Ali Bukar, Md. Shohel Sayeed

**Affiliations:** 1 Faculty of Information Science and Technology, Multimedia University, Ayer Keroh, Melaka, Malaysia; 2 Centre for Intelligent Cloud Computing, Multimedia University, Ayer Keroh, Melaka, Malaysia; University of Bucharest: Universitatea din Bucuresti, ROMANIA

## Abstract

Flash floods are severe disaster that have caused enormous damage to people, property, and the environment. Despite the conventional emphasis on technical and engineering solutions in controlling flash flood disasters, this study investigates the understudied issue of user-centric cultural viewpoints, inspired by Grid-Group Cultural Theory, and their potential impact on crisis management. The study collected 351 responses, primarily targeting adults in flood-prone areas using convenience sampling method with the goal of exploring cultural bias for feature identification of in-vehicle flash flood app. Accordingly, the research investigates the participants responses using quantitative approach which includes descriptive statistics, exploratory factor analysis, average factor, and rank scoring analysis to uncover critical user-centric cultural traits that might improve preparedness, response, and recovery activities during flash flood disasters. The findings of the study identified distinct cultural biases that impact perceptions and preferences regarding features of an in-vehicle flash flood app. By integrating Grid-Group Cultural Theory as a framework for analysis, the study highlights the importance of incorporating diverse cultural perspectives into flash flood management strategies. The result emphasizes the need to apply a holistic approach that integrates people’s knowledge and practices with technical solutions. Recommendations of features for future development of in-vehicle flash flood app is provided based on each cultural bias aligned with the theory to build more resilient communities in the face of flash flood occurrences.

## Introduction

Flash floods are disasters that occur because of excessive rainfall, rapid snowmelt, or dam failure, resulting in a quick surge of floodwater. These disasters happen quickly, with massive volumes of water flowing at amazing rates, wreaking devastation on human lives, infrastructure, and the environment [1]. Flash floods are difficult to mitigate and respond to efficiently due to their unpredictability and proclivity to overwhelm existing disaster management systems [2]. Moreover, urban flash floods regularly impede traffic flow, putting motorists and their safety at risk. Despite the authorities’ attempt to educate the public about the dangers of driving on flooded roadways, incidents of loss of life and property continue. Several variables, in addition to climate change, contribute to the occurrence of flash floods. Changes in land use, such as urbanisation and deforestation, can disrupt natural drainage patterns, resulting in greater surface runoff and decreased soil penetration. Topographic characteristics like steep slopes and small valleys can significantly speed the flow of water, increasing the danger of flash flooding. Furthermore, human activities such as building and inappropriate garbage management can clog natural drainage systems and worsen the effects of flash floods [3].

Given the complexity of flash floods and their growing frequency as a result of climate change [4], effective crisis management solutions are essential. It must be emphasised that perhaps flash floods cannot be avoided, however the likelihood and magnitude of serious detrimental impacts can be reduced [5]. Early warning systems have been put in place to identify possible flash flood occurrences, giving communities critical time to prepare and escape. Furthermore, floodplain zoning is widely used to limit land use and development in sensitive regions, therefore reducing exposure to flood threats. For instance, the Department of Irrigation and Drainage, Ministry of Natural Resources, Environment and Climate Change classifies the severity of flash flood occurrences according to the frequency of flash flood occurrence per year i.e., Priority 1 (more than five times), Priority 2 (two to four times), Priority 3 (one time) [6]. In year 2017, most locations in Malaysia are classified as Priority level 2 which reported between two to four times flash flood within a year. Six states reported more than fifty areas affected by flash floods in the same year i.e., Sarawak, Pahang, Negeri Sembilan, Pulau Pinang, Johor and Sabah.

Moreover, consideration of cultural elements in technological development is especially important in multicultural nations such as Malaysia, which has a great range of races, languages, and traditions [7]. Culture influences how people perceive and respond to flash flood threats, impacting their risk perception, coping techniques, and willingness to participate in disaster management initiatives. For example, Rahayu et al. [8] investigated the elements that contribute to the formation of social solidarity in the aftermath of Banjir Bandang disasters in Sungai Limau District, Padang Pariaman Regency. The authors concluded that despite living in the middle of many problems and flash floods, social solidarity appears to be greater among the Malamang and Batagak Kudo-kudo cultural traditions. In addition, Mohanty et al. [9] investigates the framework of early warning systems (EWS) for landslides, mudslides, and flooding in selected villages/districts in Badakhshan province. The Climate-related Disaster Community Resilience Framework (CDCRF) indicated a dwindling resilience culture among communities in the conflict-prone Badakhshan Mountain region. Despite their exposure to climate-induced disaster occurrences, the community has not been able to build coping skills. Moreover, according to Haunschild et al. [10], even though many Germans saw warning apps as necessary and valuable tools, only 17% have used one. Therefore, to allow meaningful interaction in such communities, technology must be compatible with a diverse range of cultural preferences and communication methods [3]. The community should be empowered to cope with and prepare for crisis or disaster to enhance overall efficacy of disaster management [11]. Technology developers may design inclusive solutions that respect users’ identities, languages, and social practices by recognizing this variety. This type of culturally sensitive technology improves accessibility, user experience, and trust, reflecting dynamic multicultural landscape and encouraging better social cohesion and digital inclusion. However, very limited research has investigated the inclusion of cultural bias as part of the proposed technology [12], yet it is thought to influence user behavior [13]. The user’s perception of new and unfamiliar technology will differ and is often influenced by personal qualities and location. Similarly, a technology based on European culture may not be suitable for consumers in Asian nations [14]. Therefore, investigating cultural perspectives in the context of Malaysia is timely.

This study contributes to the evolving field of disaster management, particularly in the context of flash flood management by providing a comprehensive understanding of the impact of cultural perspectives on flash flood management, guided by the Grid-Group Cultural Theory to better disaster management efforts. The Grid-Group Cultural Theory emphasises the influence of culture in determining perceptions, attitudes, and behaviours within communities. This theory accentuates the need of understanding cultural ideas, practises, and knowledge that impact community reactions to flash floods in the context of flash flood management. Users are more inclined to adopt in-vehicle applications when they have knowledge about the system and feel the applications are trustworthy and provide a benefit in their driving experience [14]. Thus, recognising the importance of cultural factors allows disaster management authorities to build context-specific and inclusive policies for engaging local populations in disaster preparation, response, and recovery [15, 16]. Hence, through a user-centric feature identification study, this research aims to investigate how the integration of cultural viewpoints based on the Grid-Group Cultural Theory can be utilised for identifying features for in-vehicle flash flood applications. The main objective is to identify distinct cultural biases that impact perceptions and preferences regarding features of an in-vehicle flash flood app, allowing for a holistic approach that combines knowledge and practices of the affected community with technical solutions. By better understanding how cultural aspects impact community reactions to flash floods, technology solutions providers may acquire actionable recommendations for future development of in-vehicle flash flood applications.

In the following sections, Section 2 presents a detailed assessment of the literature on flash floods, culture and how it contributes to the development of better technology or applications. This sets the stage for understanding the significance of cultural considerations in disaster management strategies. Section 3 provides an overview of the study methodology, which includes research design, research instruments, and data analysis procedures. Section 4 covers the results and discussion, which describes how score analysis is used to identify user-centric cultural elements, with an emphasis on how cultural viewpoints impact community reactions to flash floods. Furthermore, the emphasis on the relevance of Grid-Group Cultural Theory in understanding cultural elements and recommending the features of in-vehicle flash flood app based on cultural biases are also included. Finally, Section 5 summarized the study and highlighted the findings and recommendations as well as limitations of the study.

## Literature review

While different strategies have been shown to be beneficial to some extent, the rising frequency and severity of flash floods, worsened by climate change, has underlined the need for more comprehensive and context-specific disaster management techniques. The flash floods have far-reaching consequences, impacting both urban and rural areas, emphasizing the need for comprehensive and adaptable flash flood management strategies. Hence, due to countries like Malaysia’s vulnerability to frequent and violent flash floods, particularly during monsoon seasons, aggravated by urbanization and climate change consequences, an in-vehicle flash flood app is timely. To assist communities in planning for and responding to flash flood situations, the app would deliver real-time information, targeted instruction, and region-specific notifications. It would improve emergency response, promote public awareness, and respond to Malaysia’s ethnic population’s different requirements. Similar apps are also needed in other nations prone to flash floods, such as Thailand, Indonesia, the Philippines, and Bangladesh, where these technologies might help with early warnings, better disaster planning, and efficient rescue efforts.

Traditional methods to flash flood control have mostly concentrated on technical and engineering solutions, which may not adequately address the growing threats posed by climate change and other contributing variables [1]. According to Frigerio et al. [17], many previous studies have investigated the role of technology in flood control. Together with crowdsourcing, technology is proposed as strategic instruments for improving flood management. For example, the early flood warning system in Rhineland-Palatine was recently extended to incorporate an ensemble weather forecasts and online warning map [18]. Prakash et al. [19] developed FLOODWALL which is a mobile app to monitor three different remote locations in Uttarakhand, India for potential flash flood using IoT technology. The app consists of three modules namely the real-time sensors’ reporting, alert module and historical and statistical information of flood-related parameters. Geo-targeted alerts will be sent to individuals who will be affected by the predicted flood occurrence. In another study, Al Qundus et al. [20] compared historical weather data with current weather conditions to determine possible flood disasters using Support Vector Machine model. Data from sensors and Google API were acquired and transmitted to microcontrollers via a LoRaWAN network. Moreover, Mendoza-Cano et al. [21] collaborated with the local water authority and public schools in Colima as well as universities and other public institutions to develop an IoT based flood monitoring system for Colima-Villa de Álvarez in Mexico. In their work, hydrometeorological data are transmitted over 3G cellular and WiFi networks to a web application dashboard.

Additionally, Kamal et al. [22] developed a prototype using Blynk to detect, monitor, and notify affected areas in the occurrence of flash flood. Similarly, Shakib et al. [23] proposed an IoT-based smart vehicle flash flood to provide real-time updates and notification through IoT-based sensors and warnings detection to ensure safety of drivers and passengers. This approach is supported by Yogarayan et al. [24] which demonstrated that transmitting a flood detection message across a communication medium is doable and takes less than a minute to notify nearby vehicles. The authors further extended their work by using long-range communication technology and provide visual interpretation of the water level in a mobile app [25]. Additionally, mobile technology and GPS are needed to identify the user’s position and offer real-time information regarding flood conditions in the region. Amagsila et al. [26] presented a mobile application architecture to assist drivers in identifying flooded regions and classifying cars that can travel over the height of a flooded highway. The application uses GPS on the smartphone to detect the user’s position. Whenever the vehicle owner reaches the prototype’s range depending on the driver’s position, it will alert the car owner by voice about the flood situation in the region around the user, indicating whether it is passable or not.

Besides, mountainous locations are also vulnerable to floods. Therefore, Sung et al. [27] suggested an AIoT-based system which delivers real-time flood analysis, allowing authorities to monitor populations in hilly areas and provide early warning. In addition, an urban flash flood warning tool that combines a rainfall-runoff model with a flood-level map database to speed up the alert system procedure for recognizing flood-prone locations and the extent of the flood regardless of depth was proposed by Suhili et al. [1]. Using ArcGIS, the spatial flood depth distribution of the flooded region may be determined at any point throughout the storm interval. The integration of social media and mobile applications has been useful in obtaining important information from flood victims [17, 28–30]. A webGIS platform which is part of the RainBo project was developed by Villani et al. [31] to aid in the preparation of flood events with detailed data and flood risk/vulnerability maps, real-time monitoring/forecast of events via the collection of observed data from real sensors, estimated/forecasted data from hydrologic models, and qualitative data collected via a crowdsourcing app.

Remote sensing and geographic information systems stand out for their capacity to anticipate and monitor floods. Wan et al. [32] proposed an Android-based GIS-based mobile application known as the RescueApp which has a reporting system, an emergency call function, disaster notifications, and geo-visualization capabilities. These characteristics are designed to make disaster response personnel’s coordination and information transfer easier. Users can utilize the reporting tool to submit flood-related events and damage reports. The emergency call function allows users to quickly request help in an emergency circumstance. Tejassvi et al. [33] proposed the implementation of geo-location notifications to send SMS information about flood conditions in real time. The authors also proposed to incorporate geo-visualization tools which enable mobile users to view and receive weather and location information during the flood. Similarly, Kouadio and Douvinet [34] employ smartphone technology for geo-location and emergency network access, which allows users to be promptly warned when a flood occurs and allows them to share their whereabouts with others. The application offers the ability to report flood conditions in real time, providing crucial data on current flood conditions to emergency responders and other stakeholders as well as provide users personalized alerts based on their location.

According to Frigerio et al. [17], a mobile app known as the MAPPERS app demonstrates how technology and crowdsourcing may help improve flood resistance. The app has capabilities to allow users to record water levels, which aids in flood response. The app also aids flood preparation by providing evacuation routes and supplies. Users can use the location-based functionality to report concerns such as broken infrastructure, communicating important issues and request assistance. Recently, Costache et al. [2] evaluated the effectiveness of several algorithms and their ensembles for mapping flash flood susceptibility. The study provides insights on how to map flash flood vulnerability using multi-criteria decision-making approaches and bivariate statistics. These machine learning approaches help to identify locations that are more vulnerable to flash floods. Notably, the kNN-AHP ensemble model improved the precision and dependability of flash flood susceptibility mapping.

Another group of researchers focused on incorporating volunteered geographic information (VGI) into disaster risk management systems, specifically for flash floods. The fundamental understanding about the concept of volunteered geographic information (VGI) and its applications in disaster management (DM) of flood-related occurrences is discussed by Arapostathis et al. [35]. User-contributed geographic details such as text, photos, and GPS coordinates are used in VGI as supplements of traditional sources such as maps and satellite photos. VGI technology provides real-time data on the location and intensity of floods. The availability of VGI, such as social media and crowdsourced data, enabled researchers to monitor and simulate floods. Alyaquot et al. [36] compared synthesized water depth to VGI modalities with remote sensing. The study indicates that there are considerable disparities in the geographical and temporal distribution of VGI modalities. Lowrie et al. [37] evaluated the usefulness of data from Waze as supplements of flash flood VGI. The association of Waze flood reports with well-known flood observations and maps, such as the National Flood Hazard Layer (NFHL), high watermarks, and low water crossings data inventory was also explored by Safaei-Moghadam et al. [38]. Additionally, a unique three-step approach for extracting and mapping flood severity data using pre-trained convolutional neural networks was proposed by Feng et al. [39] to demonstrate that VGI may be utilized as a supplement to remote sensing data for flood extent mapping and is beneficial, particularly in metropolitan regions where infrastructure frequently obstructs water.

**Fig 1:**
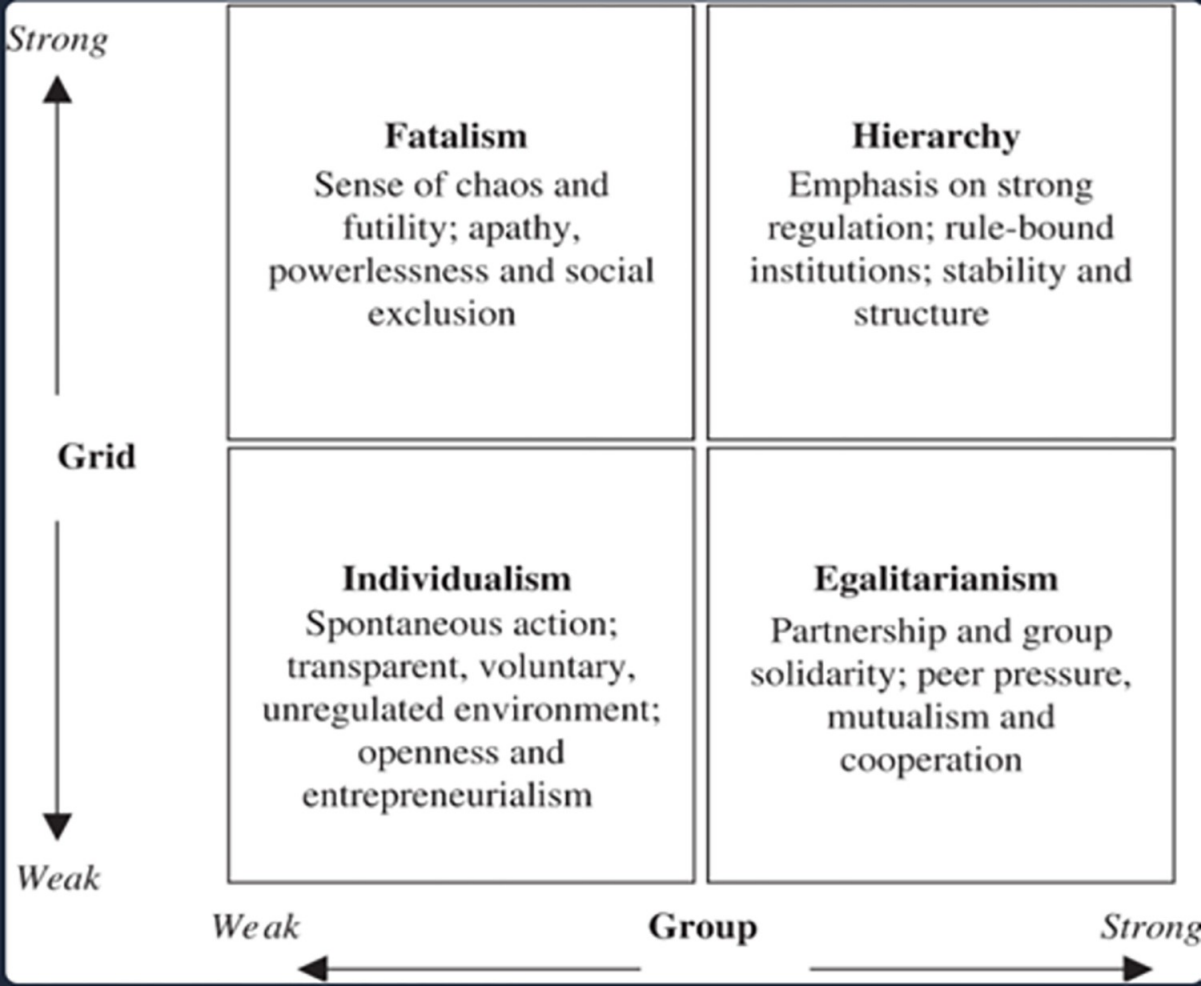
Dimensions of the Grid-Group Cultural Theory.

Based on the previous work related to technology and engineering solutions for flash flood control, there is an absence of cultural considerations in the solutions. According to Parsons and Lykins [40], an individual’s perception towards risk is influenced by his or her cultural world views or cultural biases. The authors investigated the influence of cultural biases towards people’s risk perception during disaster including flood, bushfire, storm and earthquake in Australia. Cultural biases may trigger their reaction or response during a disaster such as flash flood [41]. This knowledge provides the rationale behind investigating user-centric cultural aspects in the development of in-vehicle flash flood apps. In crisis communication, public resilience of impactful social media crisis communication in flooding emergencies was investigated through the lens of social media crisis communication theory [42]. This study holds that social interaction is a determining factor in the aftermath of crisis. Moreover, various studies have emphasized the significance of this element [29, 43–45]. While the Grid-Group Cultural Theory holds that culture influences people’s perceptions, attitudes, and behaviors within cultures. This theory emphasizes the role of cultural ideas and practices in shaping human interactions with the environment, especially in the management of environmental risks and hazards. Since individuals can be categorized based on their orientation towards rules, hierarchy and individualism, the Grid-Group Cultural Theory dimension emphasizes the importance of cultural views in molding community reactions to flood occurrences in the context of flash flood control [15, 16] as shown in Fig 1.

Accordingly, the theory can be considered as an explanatory theory which helps to comprehend how various cultures and communities perceive and respond to risks and uncertainty [46]. When identifying features for an application, the theory provides flexibility which allows creative brainstorming among application developers. It is founded on the assumption that people in society have diverse biases and value systems that influence their attitudes and behaviors when it comes to risk. Cultural biases are classified into four kinds based on two dimensions i.e., Grid (Individualism/Hierarchy) and Group (Fatalism/Egalitarianism) [41]. The dimensions help app designers and developers tailor features to meet different cultural expectations. Individualism has few formal norms and organizations in societies with a weak grid. Individuals have greater personal freedom and autonomy, and there is more tolerance for differing ideas and behaviors. Individualism and independence are more prevalent in people living in weak grid cultures. On the contrary, hierarchy is presented in the strong grid which has higher weightage in rigorous laws, formal hierarchies, and well-defined structures. There is a significant focus on obeying authority and adhering to established rules. People who live in strong grid cultures appreciate stability, order, and uniformity [40]. Hence, app users from this dimension prefer clear instructions and structured interfaces in the app design. Moreover, fatalism refers to a society with weak group dynamics, social cohesiveness and minimal individual bonds. People who adopt a fatalistic attitude towards risk believe that outcomes are beyond their control and that they are unable to change their fate [46]. However, egalitarian involves strong group dynamics with a strong feeling of community, shared ideals, and collaborative decision-making in cultures. People who come from strong group cultures value equality and collaboration, and they may be more amenable to community-based risk management measures [40, 41]. For example, users from this dimension will expect applications with social features and collaboration tools for them to engage. Overall, these four cultural biases shape how individuals perceive and respond to risks and uncertainties, resulting in various cultural worldviews, upon which this study is grounded. By utilizing the grid and group dimensions of the Grid-Group Cultural Theory, it will be possible to determine app features which align with cultural norms.

## Materials and methods

### Study design

This study sought to discover user-centric cultural aspects essential to flash flood management, as well as to comprehend how cultural viewpoints impact community reactions to flash floods. This study’s participants were chosen utilising a convenience and snowball sampling method. Participants were chosen from the authors’ networks based on their availability and willingness to participate using convenience sampling. Subsequently, the participants were then asked to introduce other potential participants from their social networks who have experience with flash flood occurrence. A Google form link was sent through numerous methods, including social media platforms, and email lists, to attract participation from 5^th^ January 2023 until 31^st^ March 2023. Throughout the study, ethical rules were scrupulously followed. 351 respondents gave their informed consent to participate in the study by filling out the Google form and submitting their replies. To safeguard their privacy and confidentiality, the study was authorized by the University’s Ethical Review Committee (Approval code: EA0852022). Individuals who came across the link were urged to fill out the form on their own initiative if they have direct knowledge or insights about flash flood control. While this strategy yielded useful insights from people who had direct or indirect encounters with flash floods, authors note the possible biases and limits of non-random sampling approaches. The study sought to supplement this sampling strategy with scoring analysis approach in order to achieve a full grasp of cultural viewpoints and their influence in defining disaster management strategies.

### Survey instrument

This study’s survey instrument was developed to collect quantitative data on user preferences, views, and answers related to flash flood, with a special focus on finding user-centric cultural elements. The items were derived from relevant literature on flood-related applications and cultural factors. The convergent validity of the items for each dimension (construct) was determined using the Average Variance Extracted (AVE) analysis. AVE values of higher than 0.7 verified the convergent validity of the items. The instrument was divided into two sections, i.e., Section A and B, and designed to be straightforward, succinct, and simple to use, delivering a pleasant experience for respondents. Section A gathers demographic information to understand the participants’ background. This section typically included questions about age, gender, occupation, level of education, and common types of transportation. The information helps to guarantee that the study collects various opinions from people with diverse backgrounds and experiences in flash flood management. The replies of participants in this area are kept anonymous and used solely for research purposes. Besides that, Section B focuses on examining the cultural bias of the respondents based on the Grid-Group Cultural Theory as in Table 1. The Likert scale was applied where 1- strongly disagree, 2-disagree, 3-neither agree or disagree, 4-agree and 5-strongly agree.

**Table 1:** Questionnaire items for Section B.

Cultural Bias	Questionnaire Item
Hierarchical	During a flash flood event, I prefer to receive information and instructions from government authorities.
	Strong leadership and centralized decision-making are crucial for effective crisis management.
	I believe in following established rules and guidelines during emergencies.
	I trust official sources of information more than other channels during a crisis.
Individualist	During a flash flood event, I prefer to make my own decisions based on my circumstances.
	I value flexibility in communication channels during emergencies.
	I believe in adapting to the situation rather than strictly following predefined plans.
	Personal autonomy is essential in handling flash flood risks.
Egalitarian	It is important for the community to be actively involved in crisis management decisions.
	I prefer a collaborative approach to crisis management, involving input from various stakeholders.
	Equal participation from all community members leads to better crisis outcomes.
	Collective decision-making is critical for effective flash flood response.
Fatalist	Flash floods are inevitable, and there is little we can do to prevent them.
	During a crisis, I feel that human efforts have limited impact on the situation.
	I do not believe that individual actions can significantly change the outcome of a flash flood event.
	Preparing for flash floods seems futile since they are beyond human control.

In addition, respondents were also required to identify features which they consider useful for an in-vehicle flash flood application as in Table 2. The Likert scale is also applied i.e., 1-totally not useful, 2- not useful, 3- maybe useful or not useful, 4-useful and 5-totally useful.

**Table 2:** In-vehicle flash flood app features.

ID	Features
F1	Real-time updates on areas with potential flooding.
F2	GPS-guided maps to help users avoid flooded areas.
F3	Voice command feature for hands-free operation.
F4	Educational guidance and videos on dealing with flood emergencies.
F5	In-app chat or call feature for contacting emergency responders or other users in case of emergency.
F6	Integration with social media platforms for easy sharing of alerts and updates with friends and family.
F7	Ringing system to keep users alert and awake during flood events.
F8	News feed section for sharing traffic conditions such as photos and videos.
F9	Interactive checklist to help users prepare for potential floods.
F10	Location sharing feature for emergency situations.
F11	Automated emergency contact feature that alerts designated family or friends if the user is unresponsive or in danger.

Based on the list of features, respondents were asked to rate the features they perceived useful when travelling in a flash flood prone location. The scale goes from 1-totally useless, 2-not useful, 3-sometimes useful and sometimes not, 4-useful, and 5-really useful. Then, depending on their perspective, participants were asked to evaluate the traits in order of significance, from most important (ranked as 1) to least important (ranked as 11). Accordingly, throughout the study, ethical rules were scrupulously followed. The respondents gave their informed consent to participate in the study by filling out the Google form and submitting their replies.

### Data analysis

To summarize the survey responses, the obtained data were analyzed anonymously using descriptive statistics such as frequencies and percentages. In addition, the quantitative results from the questionnaires were scored to determine the cultural bias, guided by the Grid-Group Cultural Theory. On the Likert scale, participants assessed the relevance of each questionnaire item, and average scores were computed to discover the respondents’ cultural bias which may influence their preferred features in an in-vehicle flash flood application. Identifying the desired features based on each cultural bias will contribute to potential user-centric features for new apps aimed at managing flash floods. Furthermore, the Measure of Sampling Adequacy (MSA), a component of the Kaiser-Meyer-Olkin (KMO) and Explanatory Factor Analysis (EFA) was employed to show the underlying structures of the latent factors, as employed by various studies [47–49].

We conducted an Exploratory Factor Analysis based on the responses of questionnaire items listed in Table 1 in the earlier section of this paper. Exploratory Factor Analysis (EFA) is a statistical approach for identifying underlying structures (latent factors) inside a set of observable variables (items or questions) in a dataset. It explains how these variables interact and contribute to the identified factors. The Kaiser-Meyer-Olkin (KMO) test and factor loadings are two important EFA components that help understand the results [50]. Table 3 shows the Measure of Sampling Adequacy (MSA) based on KMO test and the factor loadings contributing to each cultural bias. Factor 1 is the individualistic bias, Factor 2 is the egalitarian bias, Factor 3 is the hierarchical bias and Factor 4 is the fatalist bias. The results were generated by JASP 0.17.3, which is a free and open-source statistical analysis program developed by the University of Amsterdam.

**Table 3:** Exploratory factor analysis results.

Cultural bias	KMO test	Factor loadings			
	MSA	Factor 1	Factor 2	Factor 3	Factor 4
Overall MSA	0.748				
H1	0.697			0.999	
H2	0.691			0.790	
H3	0.750			0.801	
H4	0.642			0.443	
I1	0.765	0.838			
I2	0.761	0.939			
I3	0.769	0.542			
I4	0.860	0.605			
E1	0.749		0.926		
E2	0.890		0.566		
E3	0.736		0.727		
E4	0.747		0.689		
F1	0.532				0.715
F2	0.694				0.497
F3	0.571				0.734
F4	0.763				0.520

The Measure of Sampling Adequacy (MSA) is a component of the Kaiser-Meyer-Olkin (KMO) test that is used to determine if data is suitable for factor analysis. It assesses the extent to which the correlations between items in the questionnaire are sufficient for component analysis. An overall MSA of 0.748 indicates that the observed items are well-suited for the extraction of relevant components. This score suggests that the items share a significant amount of variation, which is critical for finding underlying constructs and can be represented by factors. This implies that the elements recovered from the study are likely to be trustworthy and interpretable, offering insights into the variables’ connections. For each item, higher MSA values indicate that the correlations between items are stronger and more suited for factor analysis, whilst lower values indicate that the correlations are weaker and may not be as effective in uncovering relevant underlying components. In addition, factor loadings are an important outcome of factor analysis because they show the degree and direction of the correlations between observable items and the underlying latent factors. In this study, the factor loadings show how much each item contributes to each factor and aid in identifying trends and structure in the study. Factor loadings with higher absolute values suggest a greater link between the items and the cultural bias.

## Results and discussions

**Fig 2:**
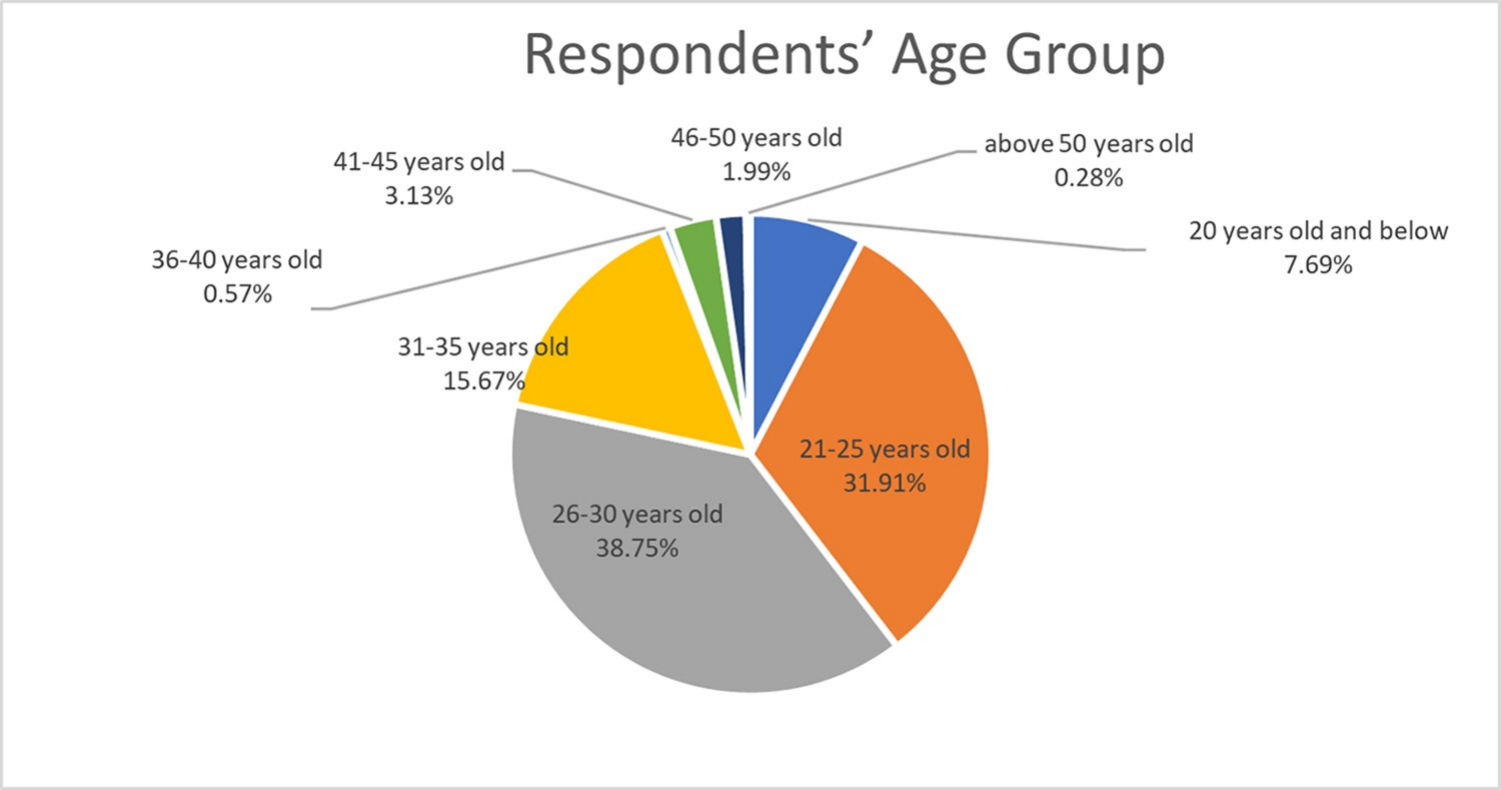
Respondents by age group.

Total respondents of the survey are 351 respondents where 68.27% are male and 31.73% are female. The age range is shown in Fig 2 with the largest group of respondents aged between 26 to 30 years old (38.75%), followed by 21 to 25 years old (31.91%) and 31 to 35 years old (15.67%). About 5.97% are mature adults i.e., above 41 years old and 7.69% are young adults i.e., 20 years old and below. Moreover, 55% of the respondents are Chinese, 37% are Malays and 8% are Indians. 33.33% of the respondents reported witnessing flash floods in the previous three years. Out of those, 67.24% had witnessed flash floods at least once, and 32.76% had witnessed many flash flood episodes.

The study revealed the presence of cultural biases among respondents, as suggested by the Grid-Group Cultural Theory using Average Factor Score Analysis [51]. These cultural biases influence how people perceive and respond to risks and crises, hence guiding the overall efficacy of flash flood management via user-centric application features. There are four distinct cultural biases, according to the theory: hierarchical, individualist, egalitarian, and fatalist. Each of these biases leads to a distinct strategy to risk management and crisis management. For instance, to encounter danger, hierarchical cultures may favor centralized power and formal institutions, whereas individualist societies may rely on individual decision-making and adaptability. Egalitarian cultures, on the other hand, may prioritize community participation and consensus-building in risk management, whereas the fatalist approach may accept fate and take a more fatalistic view to danger.

In this study, the highest factor score is individualistic (11.0716), followed by egalitarian (10.3838), hierarchical (8.4298) and fatalistic bias (4.1296). Most of the respondents prioritize personal autonomy and individual choices. Hence, app features that enable individual decision-making, such as real-time information and personalized warnings, are likely to be popular with this demographic. They may also appreciate tools that promote self-sufficiency and personalized risk management. The existence of the egalitarian bias indicates a strong preference for collaborative decision-making and shared accountability. Hence, features that emphasize community connection, group agreement, and collaborative information sharing, such as in-app chat functionality and communal notifications, may appeal to this cultural bias. While hierarchical bias has a lower score compared to individualistic and egalitarian, it indicates a preference for established systems and authority. Thus, the app may strike a balance by providing customizable features that recognize individual preferences while still giving direction and structure, appealing to the appetites of this group. Lastly, the fatalist bias recognizes a subset of respondents who believe events are affected by external forces or fate. To address this viewpoint, the app might provide a sense of security by providing clear instructions, easily available emergency contact information, and pre-defined emergency plans, assisting users in navigating emergencies even when they feel helpless. In summary, the factor analysis results provide useful insights into how these cultural biases impact choices for a flash flood app collectively and provide insight to app developers.

**Fig 3:**
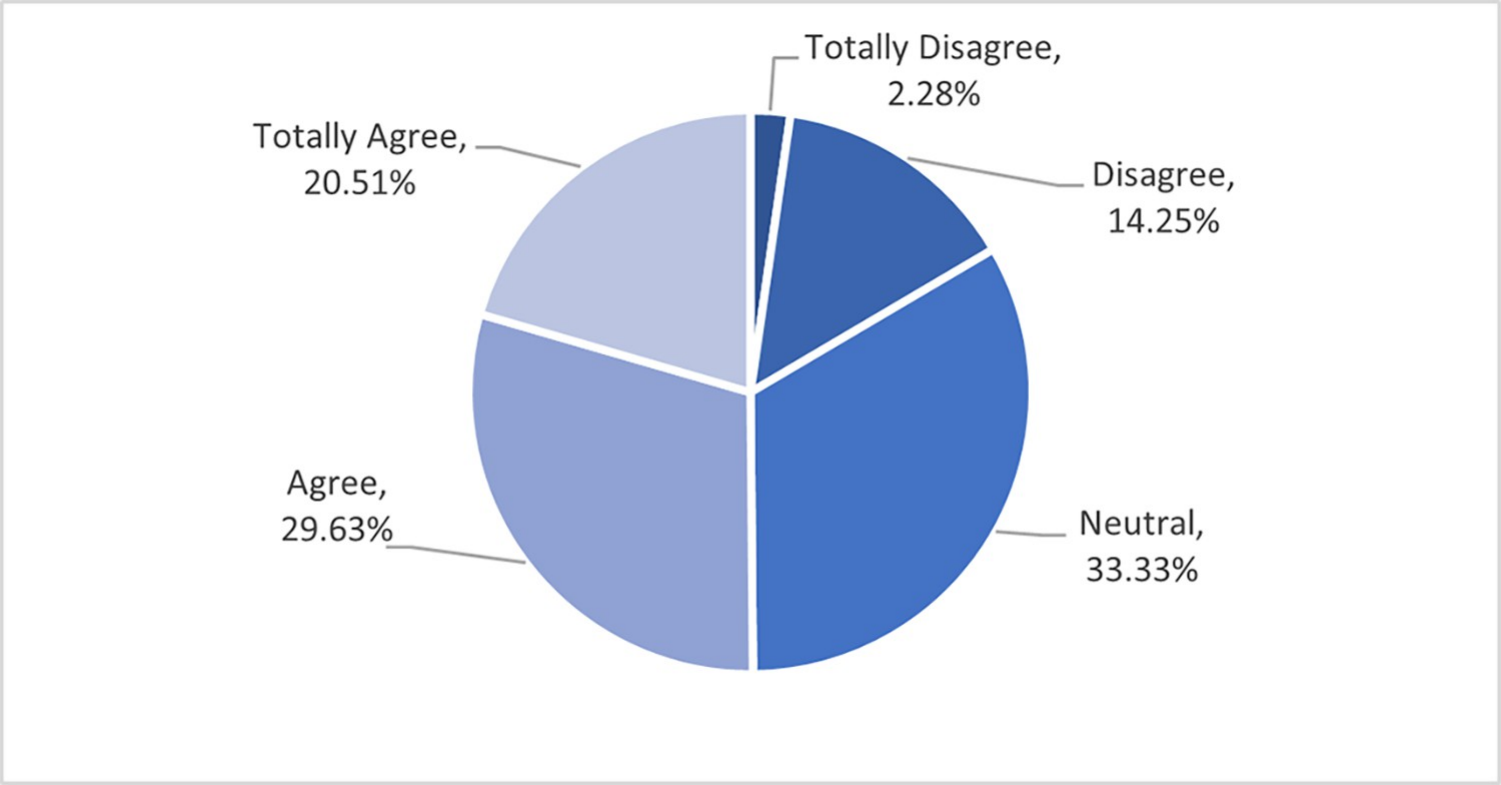
Respondents incline towards in-vehicle flash flood application.

Since technology is part of the support for crisis and risk management strategies, an in-vehicle flash flood app has been proposed to address the challenges and risk faced by drivers due to flooding including flash floods. In this study, almost 50% of the respondents are inclined towards in-vehicle flash flood apps including 20.51% (totally agree) and 29.63% (agree). Only 2.58% expressed their total disagreement with the app. Fig 3 illustrates the respondents’ responses to this item.

**Fig 4:**
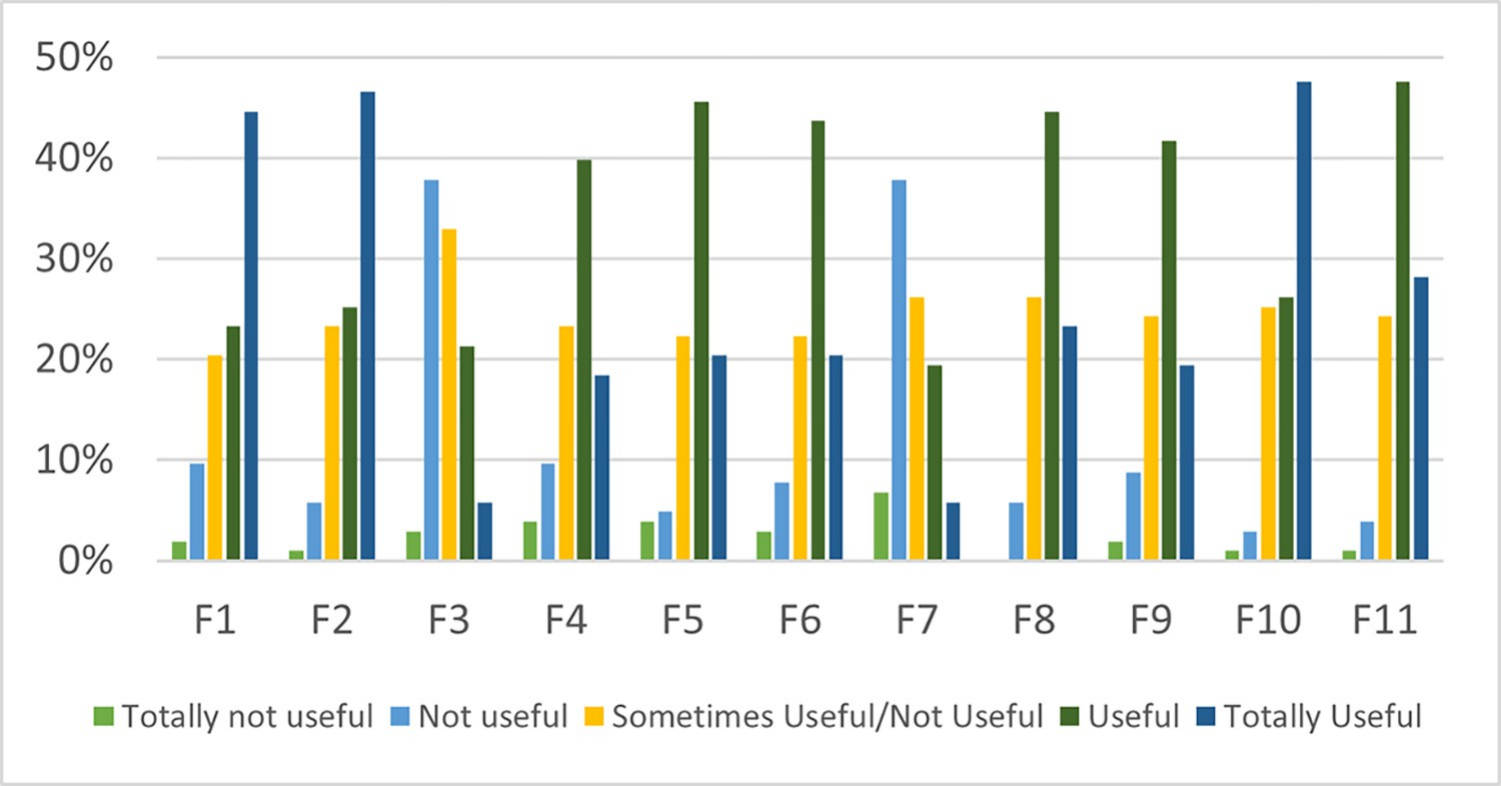
Features perceived as useful by respondents.

Based on this study also, we found that the respondents felt that there are three features which are totally useful to have in an in-vehicle flash flood app i.e., F1 (real-time updates on areas with potential flooding), F2 (GPS-guided maps to help users avoid flooded areas) and F10 (location sharing feature for emergency situations). These features were rated “Totally Useful” by 40–50% of the respondents. On the other hand, F3 (hands-free voice command function) and F7 (ringing mechanism to keep people attentive and awake during flood emergencies) were evaluated as "Not Useful" by 38% of the respondents. This response suggests that drivers are likely to remain attentive and focused during flood emergencies and therefore might not find these features necessary. Drivers often have a strong grasp on the steering wheel and heightened focus to ensure the safety of themselves and their passengers. Fig 4 illustrates the findings.

Furthermore, the rank analysis method was performed to determine which features are considered important by the respondents. The average rank for each feature was calculated. The lower the average rank, the more important the feature is considered by the respondents. The respondents’ feature ratings based on their perceived usefulness may be attributed to their cultural bias. We summarized our results in Table 4. Respondents who rated real-time updates on areas with potential flooding (F1) as "Totally Useful" (45%) may be seeking accurate updates for making well-informed decisions, which is consistent with fatalist bias tendency. Those who see it as "Useful" (23%) may value the feature’s practicality, which aligns with an individualist preference for personalized tools for handling flood conditions. Respondents who find it "Sometimes Useful/Not Useful" (20%) may be more community-focused, equating the feature with egalitarian bias.

**Table 4:** Feature importance and relations to Grid-Group Theory’s cultural bias.

ID	Rank	Feature Importance	Average Rank Score	Hierarchical	Individualistic	Egalitarian	Fatalist
F1	1	Real-time updates on areas with potential flooding.	3.72	√	√		√
F2	2	GPS-guided maps to help users avoid flooded areas.	4.23		√		
F3	3	Voice command feature for hands-free operation.	5.28		√		
F4	5	Educational guidance and videos on dealing with flood emergencies.	5.87	√		√	
F5	4	In-app chat or call feature for contacting emergency responders or other users in case of emergency.	5.47				√
F6	6	Integration with social media platforms for easy sharing of alerts and updates with friends and family.	6.19		√	√	
F7	7	Ringing system to keep users alert and awake during flood events.	6.39				√
F8	8	News feed section for sharing traffic conditions such as photos and videos.	6.70	√	√	√	
F9	11	Interactive checklist to help users prepare for potential floods.	7.37	√	√		
F10	9	Location sharing feature for emergency situations.	7.04			√	√
F11	10	Automated emergency contact feature that alerts designated family or friends if the user is unresponsive or in danger.	7.19			√	√

Moreover, GPS-guided maps to avoid flooded areas (F2) are commonly suggested in the literature. This study found that a rating of "Totally Useful" (47%) may suggest a strong individualist preference for personalized navigation solutions that correspond with their goal for personal autonomy. "Useful" ratings (25%) may come from respondents who value the feature’s practicality while making judgements based on their hierarchical preference for organized advice. "Sometimes Useful/Not Useful" scores (23%), which suit an egalitarian mindset, may suggest a need for community involvement in navigating floods. A high "Totally Useful" rating (6%) of the voice command feature for hands-free operation (F3) might be attributed to the ease of the function, which appeals to the individualist bias by allowing users to browse hands-free according to their own preferences. A "Useful" rating (21%) may come from individuals who view the feature’s utility, maybe in accordance with a hierarchical preference for efficient and authoritative tools. "Sometimes Useful/Not Useful" scores (33%) may indicate that this function isn’t always aligned with an egalitarian or community-focused perspective.

Besides, respondents who felt that educational guidance and videos on dealing with flood emergencies (F4) are "Totally Useful" (18%) may have appreciated its informative nature, which responds to the individualist bias by giving personalized assistance in times of emergency. Those who see it as "Useful" (40%) may value its practicality, which aligns with a hierarchical preference for organized and expert-driven instruction. "Sometimes Useful/Not Useful" scores (23%) may represent an egalitarian bias with community-focused mindset demanding equitable shared educational resources. "Totally Useful" ratings (20%) of in-app chat or call feature for contacting emergency responders or other users (F5) may suggest that consumers with a fatalist attitude prefer direct contact for crises, which corresponds to their demand for practical answers. Individualist respondents may like the feature’s personal interaction possibilities based on their "useful" ratings (46%). Those who find it "Sometimes Useful/Not Useful" (22%) may like the community connection part, which resonates with an egalitarian viewpoint.

The individualistic users who value personal expression and communication may be reflected in "Totally Useful" ratings (20%) of the integration with social media platforms for easy sharing (F6). "Useful" ratings (44%) may suggest a preference for the feature’s utility, which is consistent with a hierarchical preference for established communication routes. "Sometimes Useful/Not Useful" ratings (22%) may indicate that consumers benefit from community contact on occasion, which aligns with an egalitarian tendency. Furthermore, during flood conditions, a ringing mechanism is used to keep drivers attentive and awake (F7). A "Totally Useful" rating (6%) may suggest that respondents with a fatalist bias find the ringing system useful for remaining cautious amid emergencies, which corresponds to their demand for practical answers. Individualist respondents may enjoy personalized reminders that keep them interested and aware, as seen by "useful" scores (19%). The "Sometimes Useful/Not Useful" scores (26%) may indicate an egalitarian viewpoint, recognizing the common experience of remaining awake during flood situations.

Individualistic respondents who prefer real-time, personalized information regarding traffic conditions and road closures may provide "Totally Useful" ratings (23%) for the news feed section for sharing traffic conditions (F8) feature. A rating of "Useful" (45%) indicates that most respondents think the feature is useful for getting organized and official traffic reports which demonstrate hierarchical bias. "Sometimes Useful/Not Useful" scores (24%) may represent an egalitarian viewpoint, with shared traffic information valued as a common resource. Respondents who are individualistic bias and rate "Totally Useful" (19%) may consider the interactive checklist to help users prepare for potential floods (F9) useful in supporting their flood preparations. Those who are hierarchical bias felt that the checklist may be useful to systematically prepare for potential floods, as indicated by "Useful" ratings (42%). "Sometimes Useful/Not Useful" ratings (24%) may indicate an egalitarian bias that values collective resources for flood preparation.

Fatalist bias values the location sharing option i.e., location sharing feature for emergency situations (F10) for speedy aid during crises, which aligns with their need for practical solutions. This is indicated by the "Totally Useful" ratings (48%) of the feature "Useful" ratings (26%) may be provided by individualist respondents who value personalized communication during emergencies. In addition, an egalitarian bias may find value in sharing location information during crises and rated the feature as "Sometimes Useful/Not Useful" (25%). Another feature that is valued by a fatalist bias is the automated emergency contact feature that alerts designated family or friends if the user is unresponsive or in danger (F11) with "Totally Useful" ratings of 28%. Individualist bias responders may enjoy the personal autonomy and convenience afforded by this feature, as seen by "Useful" ratings (48%). Ratings of "Sometimes Useful/Not Useful" (24%) may represent an egalitarian bias that values the common experience of automated emergency calls during dangerous situations.

We acquired a better grasp of how user-centric features for in-vehicle flash flood application resonate with distinct cultural biases by linking the survey findings to the Grid-Group Cultural Theory. Recognizing these biases while developing and implementing new apps will assist assure increased acceptability and efficacy among broad groups of stakeholders. Tailoring the features to each cultural bias’s requirements and preferences can lead to more complete and culturally sensitive flash flood crisis management solutions. The survey findings emphasized the need to use a user-centric approach when developing features for applications geared at flash flood disaster management. Table 5 lists our feature recommendations for in-vehicle flash flood apps based on cultural bias.

**Table 5:** Feature recommendations for in-vehicle flash flood app based on Grid-Group Cultural Theory.

Aspect	Individualism	Egalitarianism	Hierarchy	Fatalism
Route Choices	Personalized routes based on habits and comfort	Shared real-time access and updates on road conditions, flooding and traffic	Shared routes by the authorities	Personalized routes based on habits and confidence
Alerts	Customizable alerts and notifications	Community shared alerts	Real-time updates and alerts issued based on notifications from the authorities	Early warnings when approaching flood prone areas
Navigation	Personalized navigation and alternate routes	Navigation recommendation based on collective input.	Offer route recommendations endorsed by official authorities	Provide alternate route suggestions and detours based on real-time flood and traffic data
Emergency	Set personal emergency contacts preference	Provide a platform for drivers to engage in real-time discussions and polls about route choices, weather conditions, and flood-related decisions	Include official emergency protocols; Include a centralized list of emergency contacts authorities and services	Integrated emergency contacts list; Positive affirmations and empowerment; Calming audio for flood-prone areas
Preparedness	Create and store personalized emergency plans	Nearby safe spot information	Enable users to report incidents and hazards, which are then managed and addressed by designated authorities in a centralized manner.	Calming visual elements
Support	Assistance network for emergencies	Assistance exchange for emergencies	Expert commentary on safety measures	Stress-relieving breathing exercises; Motivational flood podcast
Information	Traffic updates shared by users	Collaborative route planning with others	Quick access to relevant authorities.	Self-care reminders during driving
Weather	Customised weather updates and forecasts based on preferred locations and timeframes.	Weather updates from community sharing	Shared information and updates from authorities	Provide real-time weather and water level updates based on the driver’s current location to support informed decisions while driving and notify them on the variations

## Conclusion

We used a user-centric approach in this study to explore features for in-vehicle flash flood apps. Our findings highlight three key achievements, i.e., feature identification, alignment of the features with the Grid-Group Cultural Theory and potential impact on disaster management. First, the survey identified major user-centric features with high potential for enhancing flash flood disaster management based on survey findings. Real-time warnings, interactive maps, social media integration, and community participation are all desirable features. Real-time notifications may provide users with essential information and safety advice, allowing them to respond quickly during flood situations. Interactive maps may provide vital geographic data by directing people and emergency responders to hazard zones, evacuation routes, and shelters. Social media integration may help authorities, and the public communicate and share information more effectively, building a feeling of community resilience. Residents can be empowered to actively participate in the crisis management process by giving relevant information and reporting local situations using community engagement tools. These highlighted qualities provide practical avenues for the creation of novel flash flood crisis management solutions. Developers and policymakers may create apps that are personalized to the individual requirements and preferences of stakeholders by adding these user-centric aspects into the design. These apps can transform flash flood response techniques, improve communication and coordination, and, ultimately, save lives and prevent property damage.

Second, this study recognizes the relevance of cultural components in disaster management which may lead to increased community participation, increased preparedness and response effectiveness, and the promotion of long-term recovery initiatives. The significance of developing an in-vehicle flash flood app that aligns with cultural preferences becomes evident when considering the complexity of risk communication and disaster management. The Grid-Group Cultural Theory framework emphasizes that risk communication is influenced by numerous factors that impact individuals’ decision-making processes. Cultural norms play a pivotal role in determining what information is considered credible and reliable, how individuals process and receive information, and which actions are deemed appropriate and reasonable within specific contexts. As highlighted by many cultural theory framework, individuals’ perceptions and responses to risks are shaped by their cultural biases, which affect how they interpret information, make decisions, and engage in protective behaviours. Given these difficulties, creating an in-vehicle flash flood app that takes cultural preferences into account becomes critical. The application may successfully bridge the gap between risk communication and individual perceptions by adapting its features to connect with diverse cultural biases. For users who appreciate personal autonomy, an app that respects individualistic inclinations could prioritise personalised warnings and real-time information. Similarly, an app created with an egalitarian mindset might promote collaborative knowledge exchange and group involvement. Recognising cultural factors improves the app’s usability while also generating a feeling of confidence, dependability, and relevance across varied user groups, resulting in more effective risk communication and disaster management.

Finally, the study demonstrates the potential for in-vehicle flash flood app to transform disaster management practice, prioritizing the use of app that prioritize the requirements of users. By embracing technology and incorporating user preferences, communities may be more resilient, more prepared, and safer in the face of future flash flood disasters. While our findings have important implications for managing flash floods, the convenient sampling method and response biases in self-reported data may occur. Further research and cooperation among stakeholders and developers are required to confirm and apply these results in a larger context. Moreover, the study’s limitations include potential biases in self-reported data and the geographical scope of the study area, which may limit the generalizability of the findings. Efforts were made to mitigate these limitations and provide valuable insights into user perspectives on flash flood crisis management. Finally, this user-centric feature identification research is an important step towards developing in-vehicle flash flood apps that prioritize the requirements of users. By embracing technology and incorporating user preferences, communities will be more resilient, more prepared, and safer in the face of future flash flood disasters. Nevertheless, it is critical that we continue to investigate and incorporate input from end users to constantly develop and improve these products. We may aim for more effective and lasting solutions for flash flood crisis management by developing a collaborative and user-centered approach, contributing to the overall safety and well-being of our communities.
